# Recommendations for Enhancing Oral Health Professional Education Through the Student Voice: The ADEE‐EDSA Partnership in Action

**DOI:** 10.1111/eje.13123

**Published:** 2025-06-03

**Authors:** James Field, Bogomil Sabev, Julia Davies, Jonathan Dixon, Denis Murphy, Sibylle Vital, Joshua Kennedy

**Affiliations:** ^1^ Cardiff University Cardiff UK; ^2^ European Dental Students Association Amsterdam the Netherlands; ^3^ Malmö University Malmö Sweden; ^4^ The University of Sheffield Sheffield UK; ^5^ ADEE Dublin Ireland; ^6^ Paris Cite University Paris France

**Keywords:** curriculum, education, feedback, oral health professional, student voice

## Abstract

The student voice plays a crucial role in shaping the curriculum and learning outcomes by providing unique insights into teaching and learning experiences. This paper has been written collaboratively with the Association for Dental Education in Europe (ADEE) Curriculum Taskforce and the European Dental Students Association (EDSA) following a number of years of collaborative working. The paper discusses the increasing focus on, and importance of, involving Oral Health Professional (OHP) students as partners—and ways of recognising and jointly acting on student feedback and ideas. The paper describes various collaborative projects between ADEE and EDSA and makes recommendations for areas that would merit further exploration with students and educators alike.

## 1 Background

The student voice plays a crucial role in shaping the curriculum and learning outcomes by providing unique insights into teaching and learning experiences. This paper has been written collaboratively with the Association for Dental Education in Europe (ADEE) Curriculum Taskforce and the European Dental Students Association (EDSA) following a number of years of collaborative working. The paper discusses the increasing focus on, and importance of, involving Oral Health Professional (OHP) students as partners—and ways of recognising and jointly acting on student feedback and ideas. The paper describes various collaborative projects between ADEE and EDSA, and makes recommendations for areas that would merit further exploration with students and educators alike.

## 2 The Student Voice

The student voice plays a crucial role in shaping the curriculum and learning outcomes by providing unique insights into teaching and learning experiences [1]. Even in the late 1960s, school children were asking to break away from the scholar‐academic approach to teaching; striving to engage within an educational community, where their voice is heard and they could contribute as autonomous individuals. Whilst the quotes below refer to teenage school children from over 50 years ago [2], they are included to emphasise the fact that this drive is not new, nor is it isolated or unique to students at the point of arrival in higher or further education. The students are explaining what they would like to see from their educational environment—and it is important to note, given the numbers of students now normally progressing into further and higher education, that these matters have largely not been addressed adequately in either sector:

A school, where the teacher is regarded as a friend, and yet respected. Where the barrier of the desk is overcome, and learning is a series of discussions and experiments; where the formidable word ‘lesson’ is incomprehensible … and yet we learn more willingly than before. Suzy, 17

The fact is, that school contains two societies—that of the pupils, and that of the teachers. Whilst this state of affairs exists, it is difficult for each party to understand the other, and therefore respect wears thin. If the two parties could mix at all times … then I feel sure that both sides would begin to show a certain respect for each other. After all, by discussing problems of method with the pupils themselves, and generally joining in with their society, a greater understanding between the two generations will occur. Richard, 17

Whilst the concept of the ‘student voice’ in Higher Education is not new, it has gained more traction in recent years. The importance of the student voice has been discussed in higher education since the early 1990s, when educators started to move away from the more scholarly‐academic approaches [3] to curriculum development and delivery—and started to give greater consideration to the lived experiences of their students. This paradigm shift could be considered a key part of their duties as ‘academic citizens’ [4]; academics who demonstrate a love for the discipline, but at the same time, a deep consideration for their students. Cook‐Sather [5] proposes that young people have ‘unique perspectives on learning’ and that ‘their insights warrant not only the attention, but also the responses, of adults’. As such, it is expected that they should be afforded opportunities to actively shape their education.

Much can be learned from what students were asking for—and since that time, there have been multiple reviews and exemplars of the student voice within the Higher Education environment; including case studies of good practice [6]. A report from AdvanceHE in the U.K. concluded that student voice initiatives not only yielded the creation of materials and processes for use at that time, but also provided ongoing benefits to students and staff. These areas of improved development included:

- Personal growth (including building confidence, leadership and communication skills).
- Increased transparency (in terms of time and dedication to developing and planning learning activities).
- Increased opportunities for student‐staff dialogue and a building of ‘community’.
- Stronger staff‐student relationships, which led to a greater sense of ‘belonging’.

More recently, the concept of ‘Students as partners’ was proposed in higher education. Students are considered co‐creators of knowledge and encouraged to actively collaborate with faculty, notably in curriculum design. The objectives are not only to enrich students' experiences but also provide them skills for their future careers [7, 8].

## 3 The Student Voice in Oral Health Professional Education

The delivery of Oral Health Education is complex, involving a range of clinical and workplace‐based learning environments, contrasting student experiences and transitions, and presenting distinct challenges relating to student progress and student wellbeing [9, 10, 11]. In this regard, it could be argued that the student voice plays an even more important role. However, the student voice is not a simple construct. It requires both pragmatic application and critical reflection to ensure that it meaningfully informs educational practices. Unless student ideas are translated into action *and* students informed of the outcomes, then the process is at risk of becoming a ‘tokenistic’ exercise [12]. The complexity underscores the need for structured, ongoing dialogue between students and educators, ensuring that student contributions translate into tangible, actionable improvements.

New models of teaching and learning have embraced collaborative approaches, recognising students as key stakeholders in shaping the future of dental education [13]. These developments highlight a broader shift towards more inclusive, responsive educational frameworks—ones that not only acknowledge but actively integrate the lived experiences of students in the pursuit of excellence in dental training. It is positive to see that the student voice is increasingly being considered as a matter of course for a number of curriculum development projects and processes in Oral Health Professional Education [14, 15, 16, 17, 18].

## 4 The ADEE EDSA Partnership

ADEE [19] is the organisation that represents the interests of Oral Health Professional (OHP) educators across Europe. They collaborate closely with EDSA [20]. For nearly five decades, ADEE has built a comprehensive network of dental educators and has led strategies to harmonise and develop OHP education through taskforces, consultations, annual meetings, and Special Interest Group (SIG) activity [21]. Since it was founded in 1988, EDSA has grown in size and influence, now representing over 70 000 dental students across 35 member countries. Aligned with the core values of the ADEE, EDSA advocates for dental students by informing them about relevant policies and defending their interests in dental education and practice [22].

With mutual recognition of qualifications across EU member states, it is important to recognise the diversity of students' educational backgrounds, the variation in curriculum content and design, and the lived experiences of the students who are studying OHP programmes. The collaboration offers a unique opportunity to co‐create best practice while addressing inherent curricular differences and deficiencies [21]. Additionally, valuing student input can elevate the quality of training, helping programmes stay current with advancements and align with the practical challenges students will encounter in their careers.

Collaboratively, the ADEE GED taskforce and EDSA have considered the concept of the student voice across a number of domains. These are discussed below and, in turn, some recommendations for good practice are made. While many schools will already be able to provide evidence of these, they may act as a benchmark across the educational community. It is hoped that these exemplars can be used both intra, but also trans‐professionally by educators and students alike. The paper concludes with suggested areas that would merit further exploration with students and educators that are currently under‐reported in the literature.

## 5 Approaches to Capturing the Student Voice

Student voice can be gathered through both informal and formal approaches. Informal methods, such as staff–student events and everyday interactions, encourage open dialogue and immediate responsiveness. The importance of these instances should not be underestimated; and it is important that schools provide opportunity for students to engage informally with staff. Formal mechanisms, such as course evaluations, formal teacher feedback, and longitudinal monitoring (at various points over time), can provide structured (and often consistently regulated) insights into student experiences [23].

Institutional frameworks, such as staff–student meetings, student representation on committees, and dedicated support roles, should ensure that feedback informs decision‐making. By integrating these approaches, Oral Health Professional (OHP) education can actively embed the student voice into curriculum development and institutional policy [24]. In turn, the authors believe that students should see value in the transactional nature of the student voice—reinforcing a degree of mutual respect between both parties. A culture of openness can be created within a school when the students and educators value a shared educational community. This can be developed through a series of regular informal events with teachers, where any perceived barriers between the two ‘communities’ can be overcome.

## 6 Impact of Student Voice on Curriculum and Learning Outcomes

As discussed above, the student voice plays a crucial role in shaping the curriculum and learning outcomes by providing unique insights into teaching and learning experiences [1]. Advocates argue that the student voice initiatives serve as pedagogical improvement strategies, ensuring that curricula evolve in response to student needs. Meaningful engagement with students fosters active participation, positioning them as partners in the learning process. Further, curriculum changes—particularly those related to assessment—can impact student anxiety and academic performance [25]. Thoughtful integration of student feedback can help mitigate such challenges, ensuring that adjustments support rather than hinder learning outcomes. Additionally, research suggests that an educator's active involvement and enthusiasm contribute significantly to student satisfaction and engagement [26].

Many initiatives centre on gathering student opinions about their learning, but stop short of democratic student participation in decision‐making [12]. This shortfall underscores the need for more inclusive strategies—moving beyond traditional feedback channels to ensure students are actively involved in shaping educational priorities, resource allocation, and long‐term planning. By viewing students not just as evaluators of existing frameworks but also as co‐creators of the learning process, OHP programmes can better align with the evolving needs of both learners and the dental profession.

## 7 Challenges and Barriers to Effective Student Engagement, Feedback and Dialogue

Despite the widespread recognition that student voice is essential to adaptive and inclusive OHP education, several challenges persist. One prominent issue is the difficulty of closing the feedback loop—collecting student input but failing to translate it into concrete changes or outcomes [12]. Whilst evaluations, focus groups, and ongoing consultations can capture several student perspectives, these processes risk becoming superficial if educators do not systematically analyse and address the issues. At the same time, it is important for students to appreciate that their voice is present at one moment in time, which forms part of a longer timeline of student opinion; often student suggestions can cause educational approaches and systems to rotate back and forth as cohorts of students move through the system. Further, students must appreciate that there are often a mix of conflicting student voices at any one time—and it should not be the case that those who shout the loudest or make the biggest fuss determine the final course of action or drive for change.

The construct of the student voice is further complicated by the fact that students from varied educational, ethnic, and social backgrounds may arrive with different expectations of, and perspectives on, their educational experiences [27]—including expectations on the value, and utility, of their student input [28]. Research in Dental student suggests, for instance, that individuals of different genders may perceive their educational environments differently [29]. Additionally, individual student motivations and emotional needs can affect the way feedback is given and received, which has implications for how we engage with our students in different environments. This concept carries further in OHP education, with students often encountering multiple clinical teachers across the duration of their programmes. This can result in hundreds of episodes of direct feedback—and depending on how the parties interact with each other, can significantly affect the student experience [16]. Achieving this balance—sometimes referred to as ‘quality of influence’—requires the educator to flexibly tailor instruction, support, and feedback to the needs of smaller groups or individual learners within larger cohorts [16, 30, 31].

Concerns about the validity and reliability of student feedback also pose a significant challenge. Critics argue that students, particularly early in their programs, may not have the disciplinary depth to evaluate fully the currency or dimensions of course materials, nor the pedagogical rationale behind certain teaching methods [12]. Indeed, some question the extent to which student evaluations of teaching alone can serve as a primary measure of educational quality [12]. This scepticism is not unfounded, as the complexity of curriculum design, clinical requirements, and professional standards may surpass the average student's immediate understanding or experience. Nonetheless, this should not prevent schools from engaging with the process of ‘appreciative enquiry’ [32] which seeks students' opinions on how to build on existing good practices.

## 8 Transitioning to Further or Higher Education

As we strive to support our students in the best possible ways, it becomes increasingly apparent that the way in which students *transition* into OHP programmes is critically important. Of course, this transition does not end at the point of the student arriving and beginning their studies—but it is a phase in which students attempt to adjust and cope with their new educational and social environments [33]. The biggest change for most students is in adjusting to a more scholarly‐academic and adult way of learning. Accounting for these needs poses an opportunity when developing higher education programmes—to ensure that adequate time is given at the beginning of the programme(s), to emphasise the collaborative nature of the learning environment, and the value and utility of the student voice.

## 9 Summary of Recommendations

**Recommendation 1:** The breadth of opportunities for hearing and acting on the student voice should be made clear to all parties. Schools should work with students to communicate this effectively, perhaps with examples of how comments, suggestions or concerns can be raised, and how they are likely to be processed and acted on. Often this is best done with an explainer video (a short, engaging video that explains a concept, product, or service in a clear and concise way, often using animation or other visual elements), or an infographic showing various example pathways.

**Recommendation 2:** Student representation should be sought for all reporting, development, and quality assurance processes within the School, whilst providing opportunity for individual students to feed into these processes. The former is necessary to allow the student body to develop a meaningful representation, consulting, and reporting structure, and can employ student representatives; whilst the latter is important to facilitate the voices of individuals whose voice has not been heard or represented effectively, or who feel uncomfortable engaging with the formal processes. This can be facilitated by creating separate, less formal opportunities to feedback, or by providing an online survey for students to visit individually.

**Recommendation 3:** The student opinion should be sought on any potential change to a programme of study or assessment strategy. Students should be invited to help develop and validate marking criteria or provide constructive feedback on proposals.

**Recommendation 4:** Student voice processes and pathways should explicitly address how the feedback loop will be closed—at what point (and how) students will be notified of the outcomes of their comments or feedback—and what kinds of outcomes the students should expect as a matter of course. Where possible, schools should provide a narrative to support the position taken, to help students better understand the final outcome(s).

**Recommendation 5:** Schools should train staff and students explicitly in giving and receiving effective feedback. This training should address matters such as methods of constructing and delivering feedback, peer review and critical appraisal, and important traits such as credibility, empathy, and authenticity. The whole educational community should be trained to enact equity and inclusivity and respect diversity within the staff and student body. Teachers should be trained to engage students flexibly in conversation, rather than maintaining a consistently rigid approach/pattern to communication. Students should be encouraged to openly discuss their preferences for communication with individual teachers.

**Recommendation 6:** Schools should ensure that curricula and learning outcomes are correctly positioned to optimise engagement of students at any given stage of their programme(s). Curriculum and topic leads should be familiar with relevant descriptors for qualifications frameworks and respectful of prior student educational journeys and backgrounds to ensure that students are able to work at the intended level. Students should be encouraged to feed back on the level of learning at each stage of their programme.

## 10 ADEE‐EDSA Collaborations

### 10.1 The Graduating European Dentist Curriculum

In 2017, ADEE sought representation from EDSA to collaborate on re‐defining the Graduating European Dentist (GED) curriculum (https://adee.org/graduating‐european‐dentist). This curriculum was built around a new series of Domains (1–4) and was published in 2017 with the intention of guiding best practice in OHP education across Europe [34]. This project saw the beginning of a new way of working between ADEE and EDSA involving the student voice and student co‐creation of resources. The new GED framework has proven very popular with educators, as demonstrated by the fact that the documents themselves have been cited over 400 times. They are emerging as a key reference for discussing the expectations of graduate dentists across Europe. The new approach, based on learning outcomes, is helping to facilitate better pedagogical alignment with local curricula.

### 10.2 O‐Health‐Edu

Shortly after the GED was published, EDSA were invited to participate in O‐Health‐Edu—an EU‐funded collaborative Erasmus+ project which aimed to ‘better understand the existing state of OHP education in Europe and to develop a common vision of this education’ [21]. EDSA were involved longitudinally through the project, contributing the student view to O‐Health‐Edu's numerous intellectual outputs. The project published a ‘Vision’ for OHP education across Europe. The vision aligns with the World Health Organisation milestones (2016) and resolutions (2021), and EU4Health programme (2020) objectives—and projects 20 years into the future, to 2040. This longitudinal vision takes a multi‐stakeholder perspective to deliver OHP education that acts in the best interests of both students and patients and sits within the context of a wider strategy for general health [15]. The project culminated in the development of an online data hub that was used to establish baseline data on programme structures and curriculum practices across Europe—providing public access to data for educators, regulators, existing students and prospective students alike [35, 36].

### 10.3 Embedding Research Into the Undergraduate Curriculum

In 2023, a joint exploratory study between ADEE and EDSA examined European OHP students' views on integrating research into undergraduate curricula [17]. This revealed strong support from students at all stages of their programmes for research training, which was seen as essential in order to practice robust evidence‐based clinical practice. Students expressed enthusiasm for flexible, pan‐European research opportunities and for clear and explicit learning outcomes relating to research. ADEE and EDSA organised workshops with the International Association for Dental Research (IADR) to explore further integration of research into education. The first workshop, held at the 2022 IADR General Session in China, highlighted consensus among educators on the value of undergraduate research projects, despite barriers such as limited supervision and curriculum constraints. Following the publication of the work by EDSA, ADEE's curriculum taskforce committed to embedding research as a core element within the GED curriculum [37].

### 10.4 Embedding Environmental Sustainability Into the Undergraduate Curriculum

EDSA have been actively involved in a longitudinal project to explore and embed environmental sustainability within the OHP curriculum. This project involved a series of collaborative discussions, surveys, workshops, and consensus statements, largely overseen by the ADEE ‘Sustainability in Dentistry’ special‐interest group. As a result, the group have now published three consensus papers on strategies to embed Environmental Sustainability in OHP curricula [38, 39, 40]. These guidance documents have also informed plans to incorporate the student voice in local curriculum development initiatives to embed environmental sustain‐ability within individual institutions [41, 42].

In 2023, in partnership with industry, ADEE and EDSA launched the ‘Practice Green’ Awards, promoting sustainability within both educational and clinical practice settings [43]. The award provides ADEE member institutions with recognition across three specific areas of focus to showcase and highlight their sustainability work—Campus initiatives, Curriculum initiatives, and Procurement and Produce use initiatives.

## 11 Current and Future Collaborative Projects

The ongoing and future collaborations between ADEE and EDSA will remain dedicated to ensuring that the curriculum for European undergraduate OHP education stays current and aligned with best academic practices. Working together, the two organisations are able to champion and lobby for new initiatives that are supported by educators and students alike.

ADEE will continue to work with EDSA when consulting on curriculum development and co‐creating resources for teaching and assessment. It is imperative that EDSA is able to give the ‘student view’ and accurately represent the voice of OHP students at all stages of their study. EDSA's unique ability to do this is what makes the ADEE‐EDSA collaborations productive and valid.

Going forward, areas for potential development include continued work on:

- Subject‐specific curricula, such as Gerodontology, as advocated by the European College of Gerodontology [44] or Cariology, as advocated for by the Alliance for a Cavity‐Free Future (ACFF) [45].
- Integrating resources into the existing GED curriculum library, ensuring that they are visible to students and educators alike.
- Standardisation of student progression across European dental schools; multi‐stakeholder opinion highlights the important role of longitudinal assessment in verifying that graduates possess the knowledge and skills required for safe practice [46].
- Co‐creation of resources to support student learning.

### 11.1 Future Focus

As each year passes, educators notice generational shifts in student preferences for learning—and the most recent examples of this discussed within the taskforce relate to preferences and expectations post‐COVID, and in the use of artificial intelligence (AI) [47, 48, 49, 50]. EDSA's involvement in this narrative is critical to ensuring that students are given the right opportunities to cater for their learning preferences and needs [51]. Often, education providers (at an institutional level) remain relatively rigid in their approaches to student learning [28]—however, it is important to begin to further explore whether the traditional models that shape teaching and learning on OHP programmes are fit for purpose. Informal student feedback reported by educators across Europe is showing that students are supportive of a shift in how they learn. As such, the authors make the following suggestions for areas that would merit further exploration with students and educators alike:

- The balance between traditional in‐person didactic delivery of information versus more dynamic, interactive methods of learning.
- The specific ways in which students are encouraged to direct their own study, and how clearly these are set against specific learning outcomes and learning resources
- The use of digital resources to support didactic teaching, which caters for a wider range of learning preferences.
- Exploring how AI can help support the exploration of knowledge‐based learning outcomes, giving the students the opportunity to further explore certain concepts in greater depth and in their own time.
- The balance of early written assessment for assessing knowledge and application, versus early case‐based discussions, for assessing the ability to interpret cases and present information.
- The balance between lower‐stakes (but longitudinal) assessments within the clinical environment, versus end‐ofof‐year point‐in‐time assessments.
- Effective integration of overarching themes such as Research, Inter‐professional Education and Sustainability.
- The inclusion and emphasis on Patient and Public Involvement and Engagement (PPIE) in how programmes are delivered, with students being given the opportunity to engage with initiatives involving the wider public.

Whilst some of these approaches are supported by contemporary narratives in the literature [52], collaborative and empirical work is needed with EDSA and students to explore these further and develop robust, contemporary evidence‐based approaches to OHP education.

By viewing students as co‐creators of knowledge and not merely passive recipients, organisations can better align European curricula with evolving global standards in healthcare [53]. Furthermore, sustained international dialogue—whether through large‐scale projects like O‐Health‐Edu or smaller targeted interventions—fosters shared responsibility for academic quality and professional enthusiasm.

## 12 Conclusion

This paper has discussed how the student voice plays a crucial role in shaping the curriculum and learning outcomes by providing unique insights into teaching and learning experiences, which educators may find valuable when designing or quality assuring their programmes. The various collaborative projects between ADEE and EDSA are discussed, and suggestions are made for further exploratory work.

## Conflicts of Interest

The authors declare no conflicts of interest.

## Data Availability

Data sharing is not applicable to this article as no new data were created or analyzed in this study.
